# Effect of Starch Microparticles on the Activation and Bactericidal Activity of Murine Alveolar Macrophages Infected with *Mycobacterium tuberculosis*

**DOI:** 10.3390/microorganisms14040800

**Published:** 2026-04-01

**Authors:** Silvia Moreno-Mendieta, Dulce Mata-Espinosa, Alejandra Barrera-Rosales, Mayra Silva-Miranda, Juan Carlos León-Contreras, Vanessa Villegas-Ruiz, Sergio Sánchez, Rogelio Hernández-Pando, Romina Rodríguez-Sanoja

**Affiliations:** 1 Departamento de Biología Molecular y Biotecnología, Instituto de Investigaciones Biomédicas, Universidad Nacional Autónoma de Mexico (UNAM), A.P. 70228, Ciudad Universitaria, Ciudad de Mexico 04510, Mexico; alejandra.barrera@iibiomedicas.unam.mx (A.B.-R.); vanessavr@iibiomedicas.unam.mx (V.V.-R.); sersan@biomedicas.unam.mx (S.S.); 2 Secretaría de Ciencia, Humanidades, Tecnología e Innovación (SECIHTI), Av. Insurgentes Sur 1582, Colonia Crédito Constructor, Ciudad de Mexico 03940, Mexico; mayra.silva@iibiomedicas.unam.mx; 3 Sección de Patología Experimental, Instituto Nacional de Ciencias Médicas y Nutrición Salvador Zubirán, Vasco de Quiroga 15, Delegación Tlalpan, Ciudad de Mexico 14080, Mexico; dulce.matae@incmnsz.mx (D.M.-E.); carlos.leonc@incmnsz.mx (J.C.L.-C.); rogelio.hernandezp@incmnsz.mx (R.H.-P.); 4 Programa de Doctorado en Ciencias Bioquímicas, Universidad Nacional Autónoma de México (UNAM), A.P. 70228, Ciudad Universitaria, Ciudad de Mexico 04510, Mexico; 5 Departamento de Inmunología, Instituto de Investigaciones Biomédicas, Universidad Nacional Autónoma de Mexico (UNAM), A.P. 70228, Ciudad Universitaria, Ciudad de Mexico 04510, Mexico

**Keywords:** *Mycobacterium tuberculosis*, starch microparticles, macrophage activation, tuberculosis vaccines

## Abstract

Tuberculosis (TB), an infectious disease caused by *Mycobacterium tuberculosis*, is a leading cause of morbidity worldwide. Along with CD4+ T lymphocytes, macrophages serve as key pillars of the immune response against this intracellular pathogen, which is essential for the prompt and effective elimination of the bacilli. Many strategies have been developed to enhance the microbicidal performance of macrophages; among them, stimulation with polymeric microparticles is one of the most promising. Exposition of infected macrophages to these microparticles may enhance non-specific innate immune mechanisms such as the promotion of phagocytosis, the induction of proinflammatory cytokines, and the production of reactive oxygen intermediates (ROI), contributing to the elimination of mycobacteria. Here, we evaluated the in vitro effect of starch microparticles (SMPs) on the activation and microbicidal activity of infected alveolar macrophages. The results demonstrate that these alpha-glucan microparticles are efficiently phagocytosed by infected macrophages and promote cellular activation, inducing reactive oxygen and nitrogen species, moderate apoptosis, and modulating cytokine and chemokine production without causing an excessive proinflammatory response. These effects contributed to the elimination of tubercle bacilli when SMPs were administered after infection, suggesting their usefulness as a post-exposure treatment that activates macrophages against the pathogen.

## 1. Introduction

Despite the many efforts to find an alternative to reinforce the variable protection conferred by BCG vaccination against TB and to eradicate the disease, it continues as one of the top 10 causes of death worldwide, causing 10.7 million new cases, 390,000 rifampicin-resistant TB cases among them, and 1.23 million deaths in 2024. Following the COVID-19 pandemic, TB has probably once again become the leading cause of death worldwide from an infectious agent [1]. While many social factors must be considered and addressed to control the disease, one of the main challenges hindering the development of improved vaccines or treatments is the microorganism itself. As a successful intracellular pathogen, *M. tuberculosis* has developed numerous sophisticated survival mechanisms, including interfering with intracellular membrane trafficking, arresting phagosome maturation in infected cells, disrupting the intranuclear immune regulatory machinery, and perturbing the ubiquitin system [2].

Among the strategies proposed to improve the host immune response against the infection, one of particular interest is the use of nano- and microparticles in vaccine and treatment formulations. The particles are not only used as delivery systems or vaccine adjuvants. Still, they may interact with professional antigen-presenting cells (APCs) of the innate immune system and exhibit properties that can be conveniently exploited to improve or accelerate cellular activation, cytokine production, and the release of microbicidal factors for disease control. Particles made from polymers such as poly (L-lactic acid) (PLA) [3], poly (Lactic-co-glycolic acid) (PLGA) [4,5], dextran [6], inulin [7,8], and chitosan [9,10] have been evaluated with promising results. Exposing infected macrophages to these microparticles enhanced cellular activation, promoting phagocytosis and mycobacterial elimination through the production of pro-inflammatory cytokines and reactive oxygen intermediates (ROI). It has also been demonstrated that particles used as platforms for drug and vaccine delivery can favor autophagy and apoptosis, thereby increasing the killing of *M. tuberculosis* and diminishing colony-forming units (CFUs) in the lungs of infected mice [11,12,13].

In our previous research on TB, we reported the use of raw starch microparticles (SMPs) as a vehicle for mucosal administration of TB antigens, resulting in the induction of antigen-specific antibodies, particularly IgG2a [14]. Then, using an experimental model of progressive pulmonary TB in BALB/c mice, we demonstrated the enhanced protective effect of the BCG vaccine when these particles were given as a nasal boost following subcutaneous vaccination, with or without immobilized antigen [15], or when they were co-administered subcutaneously with the BCG vaccine as the antigen [16]. In both cases, the survival of animals infected with H37Rv or clinical isolates was prolonged, and there was a greater reduction in lung bacillary load compared to animals that received the vaccine alone.

Despite these results and the increasing number of studies reporting in vitro immunostimulatory effects of soluble, purified alpha-glucans [17,18,19,20,21,22,23,24], reports describing immunostimulant activities of starch-based polymers [25] or modified starch-based particles [26,27] remain scarce. Consequently, this field of research is still at an early stage. In particular, for these SMPs, the background and evidence are insufficient to explain their role in resolving TB infection observed in the murine model. Notably, *M. tuberculosis* has an alpha-glucan capsule whose role in the recognition of the tubercle bacillus by antigen-presenting cells (APCs) has been demonstrated [28], as well as its roles in dendritic cell maturation, the induction of reactive oxygen species, and lymphocyte proliferation [29]. Consequently, these observations highlight the importance of alpha-glucans in initiating the protective immune response against TB, and their potential as active components to be considered in the design of an anti-tuberculous vaccine [29].

In light of our previous findings and the potential of alpha-glucans, we sought to elucidate the effect of SMPs on macrophage activation in the context of tuberculous infection. To this end, we first evaluated the viability of the mouse alveolar macrophage line MH-S after exposure to these particles and their contribution to the induction of oxygen- and/or nitrogen-reactive species, autophagy, and apoptosis. We excluded any direct antimycobacterial effect of SMPs and subsequently exposed infected and uninfected macrophages to these particles to evaluate phagocytosis, mycobactericidal activity, and cell activation. The results provide evidence of non-specific macrophage activation induced by these alpha-glucan particles, which may be beneficial for modulating the response of infected macrophages.

## 2. Materials and Methods

### 2.1. Preparation of Starch Microparticles (SMPs) and Cell Culture

SMPs (Sigma–Aldrich, St. Louis, MO, USA) were prepared as previously described [30]. The particles were dry-sterilized and washed three times with Milli-Q water to ensure that external contaminants and/or superficial ligands, other than carbohydrates, that could function as macrophage activators were removed from the suspensions. Murine alveolar macrophages line MH-S (ATCC CRL-2019, ATCC, Rockville, MD, USA) were cultured in RPMI-1640 medium (21875-034, Gibco, Grand Island, NY, USA) supplemented with 10% heat-inactivated fetal bovine serum (FBS) (16000-044, Gibco, Grand Island, NY, USA) and antibiotic-antimycotic solution 1× (10378-16, Sigma–Aldrich, St. Louis, MO, USA) and grown at 37 °C in 5% CO_2_ to 70% confluence in 75-cm^2^ cell culture flasks (Corning Inc., Corning, NY, USA). For in vitro experiments, the culture medium was removed, and 1× PBS with 1 mM EDTA was added to detach the cells. After 30 min of incubation at 37 °C in 5% CO_2,_ the cells were harvested by centrifugation (1500 rpm, 5 min) and resuspended in the corresponding medium supplemented with 10% FBS for Trypan blue exclusion counting. Cells were seeded into culture plates or flasks and grown to 70% confluence for infection and/or treatment with particles as indicated for each experiment.

### 2.2. In Vitro Cytotoxicity Assay for SMPs

The MTT toxicity assay for SMPs was performed in three macrophage lines: RAW 264.7 (ATCC TIB-71), MH-S (ATCC CRL-2019), and PMA-differentiated THP-1 (ATCC TIB-202) and in the epithelial Vero cell line (ATCC CCL-8). The cells were seeded independently on 96-well plates (Costar 3598, Corning, NY, USA) and incubated for 24 h in 100 μL RPMI-1640 medium supplemented with 10% FBS at 37 °C in 5% CO_2_ to reach a density of 2 × 10^4^/well. THP-1 cells were previously differentiated into macrophages for 24 h with phorbol 12-myristate-13-acetate (PMA) (PB139, Sigma–Aldrich, St. Louis, MO, USA) at 25 ng/mL at 37 °C in 5% CO_2_. After incubation, the plates were washed to remove PMA. For assays, new, fresh medium containing the SMPs at concentrations of 100, 200, 300, 400, 500, 600, 700, 800, 900, and 1000 μg/mL was added and incubated for 48 h at 37 °C in a 5% CO_2_ atmosphere. Then, 10 μL of MTT (3-(4,5-dimethylthiazol-2-yl)-2,5-diphenyltetrazolium bromide) (M2128, Sigma–Aldrich, St. Louis, MO, USA) (5 mg/mL in sterile PBS) was added to each well. Incubation continued for another 4 h at 37 °C according to Mosmann, 1983 [31]. The medium was removed, and 100 μL of DMSO (34869, Sigma–Aldrich, St. Louis, MO, USA) was used to solubilize the formazan. Absorbance was determined at 570 nm, and the half-maximum inhibitory concentration (IC50) was calculated as:

$$IC50=\left(1-\left(Absorbance\;sample÷Absorbance\;control\right)\right)\times 100$$

Culture medium was used for background subtraction in all groups. Cells without treatment and cells treated with DMSO 20% were included as controls. Cell viability was calculated as:

$$Cell\;viability\left(\%\right)=\left(Absorbance\;sample\times 100\right)÷\left(Absorbance\;control\right)$$

### 2.3. ROS/RNS Production Induced by SMPs

The production of oxygen and/or nitrogen reactive species induced by SMPs was determined in MH-S macrophages by fluorescence microscopy using the ROS/RNS Kit ab139473 (Abcam, Cambridge, UK). Briefly, 8 × 10^4^ cells were seeded in Nunc Lab-Tek II-4-well chamber slides (Thermo Scientific, Waltham, MA, USA) with 500 μL RPMI-1640 medium supplemented with 10% FBS, antibiotic-antimycotic solution 1×, and incubated for 24 h at 37 °C in 5% CO_2_ to reach a density of 1.6 × 10^5^ cells for the experiment. The medium was carefully removed, and cells were covered with 200 μL of the ROS/RNS 3-Plex Detection Mix and incubated for 2 h at 37 °C in 5% CO_2_ in the dark. The mix was removed, and cells were washed once with 200 μL of 1× wash buffer. SMPs (ratio 5:1, particles per cell), NO inducer L-Arginine (1 mM), and ROS inducer pyocyanin (60 μM) were added and left for incubation for 15 min. An autofluorescence control was prepared using non-stained macrophages. After incubation, the medium was carefully removed, and the testing and control cells were washed once with 1× wash buffer. The cells were overlaid with a cover slip, protected from drying, and observed under an Olympus BX51-WI fluorescence microscope (Olympus Corporation, Tokyo, Japan) coupled to a Disk-Scanning Unit, using standard excitation/emission filter sets. The images were acquired with an EM-CCD Hamamatsu C9100-02 digital camera (Hamamatsu Photonics K.K., Hamamatsu, Shizuoka, Japan) and further analyzed with Fiji software v.1.54p [32].

### 2.4. Evaluation of Autophagy and Apoptosis Induced by SMPs

The induction of autophagy and apoptosis by SMPs was evaluated in MH-S macrophages through fluorescence microscopy using the Autophagy Detection Kit ab139484 (Abcam, Cambridge, UK) and the Apoptosis/Necrosis Assay Kit ab176749 (Abcam, Cambridge, UK), respectively. Culture conditions were the same for the ROS/RNS assay. Autofluorescence and negative controls were prepared with culture medium. Positive controls were prepared following the manufacturer’s instructions. Experimental groups were prepared with an SMPs ratio of 5:1 for 3 h. After incubation, the medium was carefully removed, and the testing and control cells were washed twice with 200 μL of the corresponding assay buffer. They were then covered with 200 μL of the corresponding detection mix and incubated at 37 °C or room temperature in the dark for 30 min. Cells were washed carefully and overlaid with a cover slip for observation under an Olympus IX71 fluorescence microscope (Olympus Corporation, Tokyo, Japan), using standard excitation/emission filter sets. The images were acquired with a CCD Evolution VF color-cooled digital camera (MediaCybernetics, Rockville, MD, USA) and further analyzed with Fiji software v.1.54p [32].

### 2.5. Determination of Anti-M. tuberculosis Activity of SMPs

The susceptibility of *M. tuberculosis* to SMPs was evaluated using the colorimetric Resazurin Microtiter Assay (REMA). First, the *M. tuberculosis* H37Rv (ATCC 27294) strain was cultivated in a 7H9-glycerol-10% ADC-0.01% tyloxapol medium at 37 °C until an O.D. 600 nm ≈ 0.4 was reached. A working bacterial solution was obtained by diluting 1:25 in 7H9-ADC 10%. For the REMA, the outer wells of a 96-well plate (Costar 3599, Corning, NY, USA) were filled with 200 μL of sterile 1× PBS to prevent dehydration during the 8-day incubation. Rifampin was used as a reference antimycobacterial drug at serial two-fold dilutions (from 16 to 0.001 μg/mL) in each plate. Controls of medium and medium + *M. tuberculosis* were included to validate the plates. SMPs were evaluated at concentrations ranging from 100 μg/mL to 1000 μg/mL in independent assays performed in triplicate. Plates were incubated for six days at 37 °C in a 5% CO_2_ atmosphere. Then, 30 µL of 0.01% resazurin (weight/volume) (R7017-5G, Sigma–Aldrich, St. Louis, MO, USA) was added to each well, and the plates were incubated for an additional two days under the same conditions. A change in color from blue to pink indicated the bacterial growth, so the minimal inhibitory concentration (MIC) was defined as the lowest SMPs concentration that prevented this change in color.

### 2.6. Preparation of Bacteria for Cell Infections

The *M. tuberculosis* H37Rv (Stanford) strain was prepared as follows. From a 20% glycerol in Middlebrook 7H9 broth medium, the bacteria were striated in solid medium 7H10 supplemented with oleic-albumin-dextrose-catalase (OADC) (Difco Laboratories, Sparks, MD, USA) and incubated for 21 days at 37 °C in 5% CO_2_. After incubation, a colony was placed in Middlebrook 7H9 broth medium supplemented with OADC (Difco Laboratories, Sparks, MD, USA), 5% glycerol, and 0.05% tyloxapol (T8761-50G, Sigma–Aldrich, St. Louis, MO, USA) and incubated in a stirring incubator at 35 °C and 70 rpm until reaching the logarithmic growth phase (O.D. 600 nm ≈ 1.0). Once recovered, it was washed 3 times, aliquoted in sterile 1× PBS, and frozen at −70 °C until use. The concentration of the bacterial lot was confirmed from each thawed aliquot by colony-forming units (CFU) assay of serial dilutions on solid medium 7H10 supplemented with OADC after 21-day incubations at 37 °C in 5% CO_2_. For the cell infection experiments, frozen *M. tuberculosis* stock aliquots were thawed and then sonicated for 45 s to declump the pellet. The suspension volumes required to achieve the desired multiplicity of infection (MOI) of 5 (five bacteria per cell) were calculated from the known CFU in the bacterial suspension and prepared in the corresponding medium for each assay. The CFU numbers used for in vitro infections were confirmed in each experiment by culturing a dose on solid medium 7H10 supplemented with OADC.

### 2.7. Transmission Electron Microscopy (TEM) Demonstration of Phagocytosis

**Figure 1:**
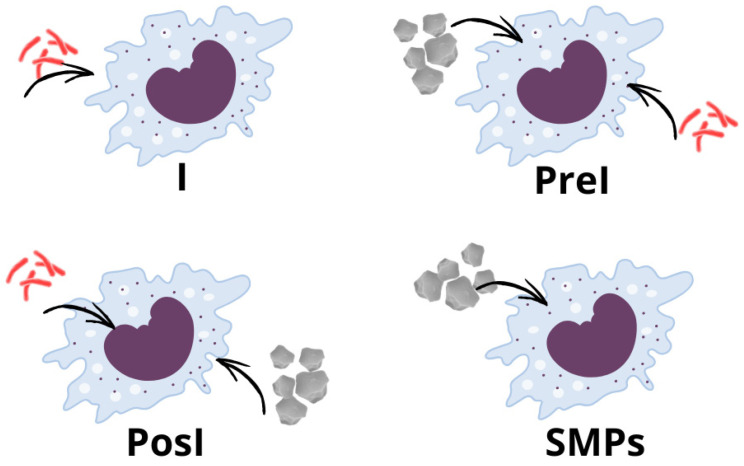
Experimental groups to evaluate the phagocytosis of *M. tuberculosis* and SMPs in MH-S alveolar macrophages. The groups were named as follows: I = Infected (MOI of 5); PreI = Pre-infection (SMPs added before infection); PosI = Post-infection (SMPs added after infection); SMPs = SMPs (5:1 ratio).

The phagocytosis of *M. tuberculosis* and SMPs by MH-S macrophages was evaluated through transmission electron microscopy (TEM). Briefly, 1.5 × 10^6^ cells were seeded in 25-cm^2^ cell culture flasks (Corning Inc., Corning, NY, USA) in 2 mL RPMI-1640 medium supplemented with 10% FBS and incubated 24 h at 37 °C in 5% CO_2_ to reach a density of 3 × 10^6^ cells for infection and/or addition of SMPs. Four experimental groups were prepared as shown in Figure 1.

The I and SMPs groups were prepared as follows. Infection with the *M. tuberculosis* H37Rv (Stanford) strain was performed at a MOI of 5. The SMPs were added at a 5:1 ratio (five particles per cell) in a final volume of 2 mL of RPMI-1640 without FBS, and incubated at 37 °C in 5% CO_2,_ shaking every 15 min for the first 3 h. After incubation, the cells were washed three times with 2 mL of 1× PBS supplemented with antibiotic-antimycotic solution 1× in the I group to remove extracellular bacilli, and three times with 2 mL of 1× PBS in the SMPs group to remove particles. Fresh RPMI-1640 supplemented with 10% FBS was added to both groups to complete the incubation for 6 and 24 h.

The PreI and PosI groups were prepared as follows. For the PreI group, the SMPs were added at a 5:1 ratio, in a final volume of 2 mL of RPMI-1640 without FBS, and incubated at 37 °C in 5% CO_2,_ shaking every 15 min for the first 3 h. After incubation, the cells were washed three times with 2 mL of 1× PBS to remove particles. Immediately after, the infection with *M. tuberculosis* H37Rv (Stanford) strain was performed with a MOI of 5, in a final volume of 2 mL of RPMI-1640 without FBS, and incubated at 37 °C in 5% CO_2,_ for three additional hours. After the incubation period (6 h in total), the cells were washed as described, and fresh, supplemented medium was added to complete the 24 h of incubation. The PosI group was prepared under the same conditions, but with the order inverted: first infecting and then administering the particles.

For ultrastructural analysis, the culture medium was removed, and cells were fixed with 2 mL of glutaraldehyde 2.5% in Cacodylate Buffer (0.15 M, pH 7.2) for 5 min at RT. Cells were scraped, transferred to 1.5 mL Eppendorf microtubes, and centrifuged for 5 min at 1500 rpm. 1 mL of fresh, cold glutaraldehyde solution was added, and the cells were fixed for 24 h at 4 °C. Post-fixation was performed using 1% Osmium Tetroxide. The samples were dehydrated using increasing concentrations of ethyl alcohol and then embedded in Spurr Low Viscosity Resin (Electron Microscopy Sciences, Fort Washington, PA, USA). The sections were made 80 nm thick in copper grids that were contrasted with 1% uranyl acetate (Electron Microscopy Sciences, Fort Washington, PA, USA) and with Lead Citrate “Reynolds”. Examination of samples was performed with a Transmission Electron Microscope FEI Tecnai G2 Spirit BioTWIN, at 80 kV (FEI Technology, Hillsboro, OR, USA).

### 2.8. Killing Assay and Nitric Oxide Production

The ability of MH-S macrophages to eliminate *M. tuberculosis* in the presence of SMPs was evaluated by CFU quantification and nitric oxide production. Cells were seeded in 24-well plates (Costar 3526, Corning, NY, USA) in RPMI-1640 medium supplemented with 10% FBS, until reaching 1.6 × 10^5^ cells/well. The I, PreI, and PosI groups were prepared as described above (Figure 1) at a MOI of 5 and a 5:1 SMPs/cell ratio, both in a final volume of 500 μL of RPMI-1640 medium without FBS, and incubated at 37 °C in 5% CO_2_.

For the I group, after the first 3 h of incubation, the cells were washed 3 times with 200 μL of PBS supplemented with antibiotic-antimycotic solution 1× to remove extracellular bacilli, and fresh medium supplemented with 10% of FBS was added to complete 24, 48, and 72 h of incubation. For the PreI and PosI groups, which completed 6 h of incubation with the strategy of adding SMPs before and after infection, respectively, the cells were washed, and fresh, supplemented medium was added to complete 24, 48, and 72 h of incubation.

The supernatants were collected at each incubation time point, filtered through a 0.22 μm filter, and stored at −70 °C until assessment. The cells were lysed for 10 min with 200 μL of 0.1% SDS, followed by the addition of 200 μL of 20% BSA (both prepared in Middlebrook 7H9 medium). Serial dilutions were performed in the same medium and plated in 7H10 medium for quantification of CFUs. The experiment was carried out in triplicate.

For nitric oxide (NO) determination, two additional groups stimulated with SMPs (at a ratio of 5:1) and lipopolysaccharide (LPS from *E. coli*, O111:B4) (L4391-1MG, Sigma–Aldrich, St. Louis, MO, USA) at 1 μg/mL/well were included. The supernatants of each group were mixed with equal amounts of Griess reagent (0.1% sulfanilamide (S9251-100G, Sigma–Aldrich, St. Louis, MO, USA) and 0.1% N-1-naphthyl-ethylenediamine (2224885G, Sigma–Aldrich, St. Louis, MO, USA) prepared in 2.5% phosphoric acid), incubated at room temperature with gentle shaking (10 rpm) for 10 min, and the absorbance was read at 540 nm. The NO_2_^−^ concentration was calculated by using sodium nitrite as a standard [33].

### 2.9. Production of Metabolites and Cytokines

To determine the level of activation of MH-S macrophages infected and/or treated with SMPs, the supernatants of killing assays were also used to quantify glucose uptake, lactate release, and cytokines. Under the same culture conditions, the groups established for metabolites determination were: Unstimulated macrophages (MH-S), I, PreI, PosI, and SMPs. For cytokine/chemokine determination, the same groups were considered, along with an additional group stimulated with LPS (1 μg/mL/well). All samples contained metabolites and cytokines/chemokines synthesized by 1.6 × 10^5^ cells/mL.

Glucose uptake and lactate release were measured in supernatants by spectrophotometry using the Glicemia Enzimática AA Kit (140060, Wiener Lab, Rosario, Argentina) and the Lactate Kit (1999975, Wiener Lab, Rosario, Argentina), respectively, according to the manufacturer’s instructions. Glucose uptake was determined by measuring the reduction in glucose in supernatants relative to RPMI 10% FBS medium and was calculated as the decrease in glucose in macrophages incubated only with complete RPMI medium. Supernatants were always harvested from the same wells.

Pro and anti-inflammatory cytokines and chemokines (IL-1β, TNF-α, IL-12p70, IL-6, IL-10, MCP-1/CCL2, MIP-1/CCL4) were evaluated by flow cytometry using the Milliplex MAP Mouse Cytokine/Chemokine Magnetic Bead Panel (MCYTOMAG-70K-07) according to the manufacturer’s instructions (Merck). The plates were analyzed on a MagPix machine (Luminex, Austin, TX, USA). The xPonent^®^ MAGPIX software v4.2 was used for the analysis of the standard Logistic 4P Weighted curves and the samples.

### 2.10. Statistical Analysis

Replicate experiments were independent, and results are presented as means ± standard error of the mean (SEM). For Multiplex analysis, deviations from expected cytokine/chemokine concentrations were calculated as *z*-scores. For most experiments, statistical analysis was carried out using one- or two-way analysis of Variance, followed by Dunnett’s or Tukey’s multiple comparison tests. Differences were considered significant at *p* ≤ 0.05. The data were analyzed using GraphPad Prism version 9.5.0 (GraphPad Software, San Diego, CA, USA).

## 3. Results

### 3.1. SMPs Did Not Exhibit Cytotoxic Effects on Macrophages or Epithelial Cells

**Figure 2:**
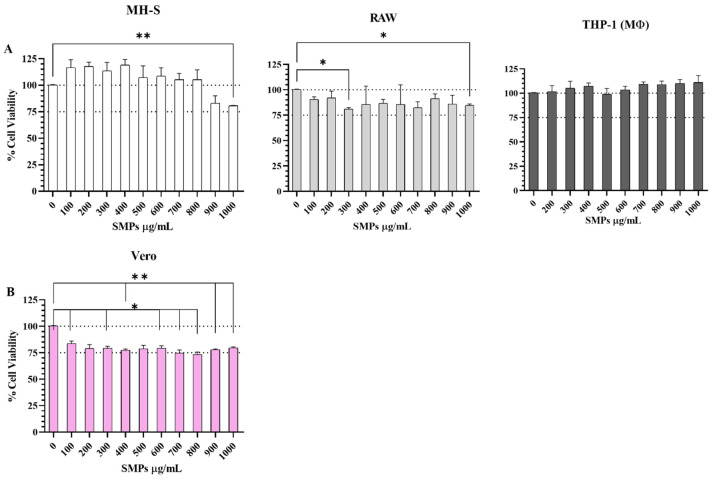
Cytotoxic effect of SMPs at 48 h determined by MTT assay. (**A**). On macrophages. (**B**). On Vero cells. Bars represent the mean ± standard error of the mean (SEM). Significance was calculated relative to the control (untreated macrophages) with a one-way ANOVA and Dunnett’s multiple comparisons test. Statistical significance * *p* ≤ 0.05, ** *p* ≤ 0.01.

The dose–response of macrophage and Vero cell lines (Figure 2A,B) to SMPs treatment was analyzed using an MTT assay (mitochondrial activity). In all cases, SMPs can be considered non-toxic. As shown in Figure 2A, the cell viability of MH-S macrophages diminished by around 25% only at high concentrations of SMPs (1000 μg/mL, *p* ≤ 0.01 relative to the control). For RAW 264.7 macrophages, cell viability was maintained between 80 and 90% at all concentrations evaluated, while for THP-1-derived macrophages, there was no reduction in cell viability. Interestingly, as shown in Figure 2B, the viability of Vero cells decreased by approximately 25% at 200 μg/mL and remained at this level at all concentrations (*p* ≤ 0.05 and *p* ≤ 0.01 relative to the control), indicating that these cells were more sensitive to the SMPs than macrophages.

### 3.2. SMPs Induced the Production of ROS/RNS in Mouse Alveolar Macrophages

**Figure 3:**
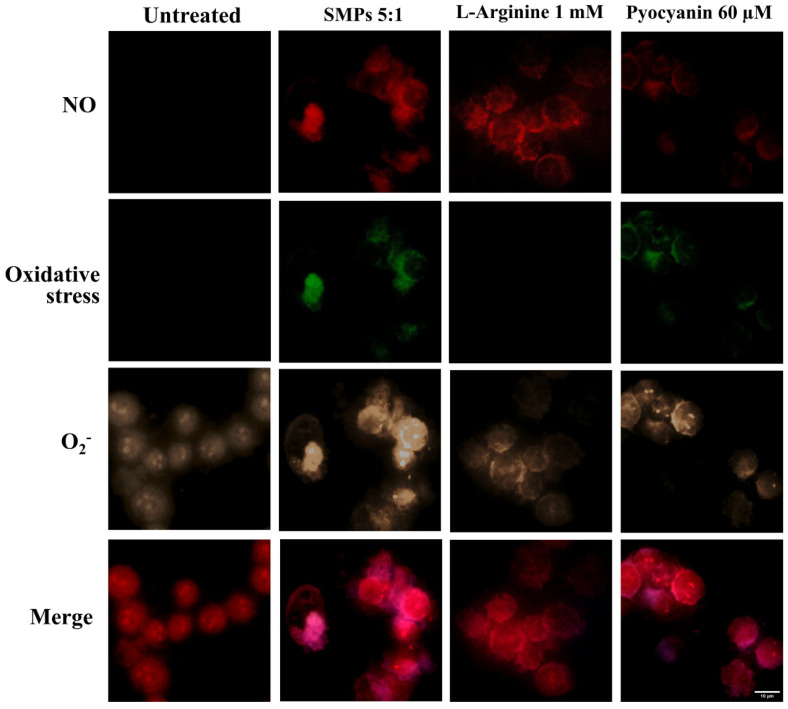
Representative fluorescence micrographs showing ROS/RNS produced by MH-S murine alveolar macrophages stimulated with SMPs (5:1 ratio for 15 min). L-Arginine (1 mM) and pyocyanin (60 µM) were used as controls for NO and ROS induction, respectively. Scale bar 10 µm. 60×. The production of NO exhibits red fluorescence, with a cytoplasmic staining pattern; the production of oxidative stress exhibits bright green fluorescence in the cytoplasm, and superoxide production exhibits bright orange nuclear staining.

To investigate the early contribution of SMPs in the induction of ROS/RNS, potentially beneficial for mycobactericidal activity, we used a kit designed for the real-time measurement of free NO and, by extension, the nitric oxide synthase (NOS) activity, as well as global levels of ROS, and specifically superoxide in living cells. As shown in Figure 3, under basal conditions, MH-S macrophages without stimulation exhibited basal superoxide (O_2_^−^) production, which appeared more intense after SMPs addition. The superoxide detection reagent reacts specifically with this molecule, generating an orange-fluorescent product, which appears sepia in the image. This basal O_2_^−^ production is essential for cellular signaling, pathogen defense, and redox balance in the lung, linked to mitochondrial respiration and NADPH oxidase activity [34,35]. SMPs addition also induced NO and ROS. The NO detection reagent yields a red-fluorescent product, while the ROS reagent reacts with a broad range of reactive species—including H_2_O_2_, peroxynitrite (ONOO^−^), and hydroxyl radicals (HO)^−^ producing a green-fluorescent product.

### 3.3. SMPs Induced Apoptosis but Not Autophagy in Mouse Alveolar Macrophages

**Figure 4:**
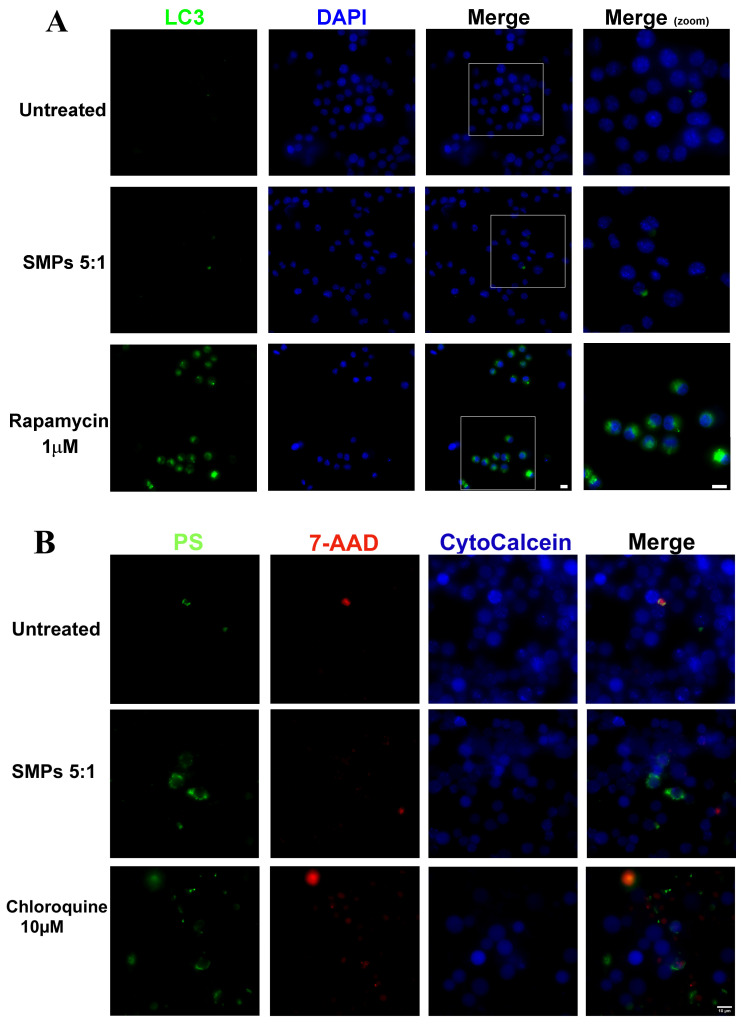
Representative fluorescence micrographs showing (**A**). Screening of autophagy in MH-S murine alveolar macrophages stimulated with SMPs (5:1 ratio for 3 h). The inducer rapamycin (1 µM for 3 h) was used as a positive control. 40×. (**B**). Screening of apoptosis in MH-S murine alveolar macrophages stimulated with SMPs (ratio 5:1 for 3 h). The inducer chloroquine (10 µM for 3 h) was used as a positive control. 60×. Scale bars 10 µm.

Since the induction of autophagy and apoptosis in infected macrophages is beneficial for inhibiting *M. tuberculosis* growth, we also investigated whether the addition of SMPs effectively induced these essential cellular activities. As shown in Figure 4A, these particles at a ratio of 5:1 did not induce autophagy in MH-S murine alveolar macrophages. In autophagic cells, the green detection reagent typically accumulates in the autophagic vesicles in the perinuclear region and the cytoplasm, colocalizing with the LC3 protein; however, we did not observe this fluorescence after addition of SMPs. In contrast, apoptotic cells were observed after the addition of SMPs (Figure 4B). The translocation of phosphatidylserine (PS) on the cell surface is a universal indicator of the initial/intermediate stages of cell apoptosis, and it was detected with the apopxin green indicator after addition of the SMPs. Cells undergoing late apoptosis and necrosis look red. These are representative images from three independent assays for each activity, each comprising several microscopic fields.

### 3.4. SMPs Did Not Exhibit Antimycobacterial Activity

To rule out any effect of SMPs on mycobacteria, we evaluated any potential inhibitory activity on the virulent *M. tuberculosis* H37Rv strain. As shown in Table 1 and compared with rifampicin (the first-line antibiotic usually indicated for TB treatment), SMPs evaluated at concentrations between 100 and 1000 μg/mL had no mycobactericidal activity. The IC50 values indicate that no amount of SMPs, up to 1 mg, was sufficient to kill 50% of the cells, suggesting that they do not exhibit cytotoxic effects on the evaluated cell lines, which are representative of epithelial and lung cells. These data agree with the results shown in Section 3.1.

### 3.5. SMPs Were Efficiently Phagocytosed by Healthy and Infected Murine Macrophages

**Figure 5:**
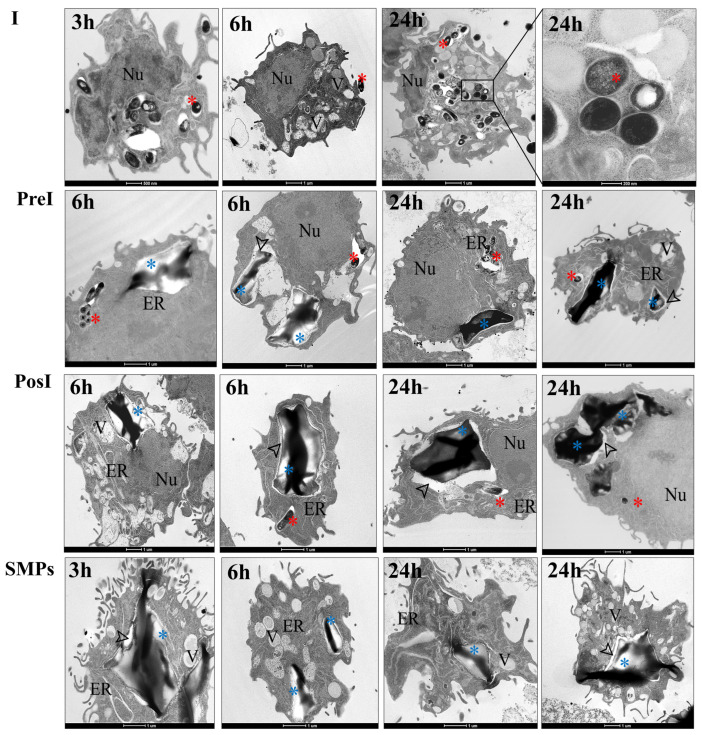
Phagocytosis of *M. tuberculosis* and SMPs by MH-S murine alveolar macrophages. Representative transmission electron micrographs showing the experimental groups: I = Infected (MOI of 5); PreI = Pre-infection (SMPs added before infection); PosI = Post-infection (SMPs added after infection); and SMPs = SMPs (5:1 ratio). In panels, structures are indicated. The red asterisks indicate the mycobacteria, which appear as electron-dense black circles or rods. The blue asterisks indicate the starch microparticles, which are observed as large, white, polyhedral granules. Black arrowheads indicate the membrane of phagosomes. Nucleus (Nu), vacuoles (V), and endoplasmic reticulum (ER) are also indicated.

The capacity of healthy and infected mouse alveolar macrophages to phagocytose SMPs was investigated (Figure 5). Together and separately, both microparticles and bacilli were efficiently phagocytosed and consistently observed within independent phagosomal compartments at all time points. Many morphological changes were also evident, including vacuoles and pseudopodia associated with cellular activation. In all groups, loose particles and/or bacteria were present too. After 24 h of incubation, dying cells, digested bacilli, and digested particles were identified in all conditions (Figure S1A).

In the I group, approximately 70% of macrophages infected at a MOI of 5 contained between 1 and 12 bacilli. At 24 h, some of the phagocytosed bacteria showed clear signs of degradation. When only SMPs were added, among 1 to 5 particles were observed inside macrophages (Figure S1B). Interestingly, in the SMPs group, empty activated macrophages were observed near those actively phagocytosing particles (Figure S1C). Some cells exhibited compact, electrodense nuclei characteristic of apoptosis (Figure S1D). The SMPs in all groups showed the morphology and central hilum (representing the center of the Maltese cross in polarized light microscopy) characteristic of starch granules, as analyzed at the ultrastructural level via TEM [36]. In both the PreI and PosI groups, the most common finding was that macrophages contained 1 and 2 SMPs, and between 2 and 10 bacilli. There was no difference observed regarding the order in which the macrophages were exposed to the SMPs or bacilli. In the PosI group, patterns of activation were also frequently observed in macrophages that had not phagocytosed but were in proximity to particles (Figure S1E). On the contrary, in the PreI group, the activation pattern was less dramatic in both empty and phagocytosing macrophages (Figure S1F).

### 3.6. Mycobacterial Growth in Murine Macrophages Was Restricted by SMPs, Depending on the Addition Strategy

**Figure 6:**
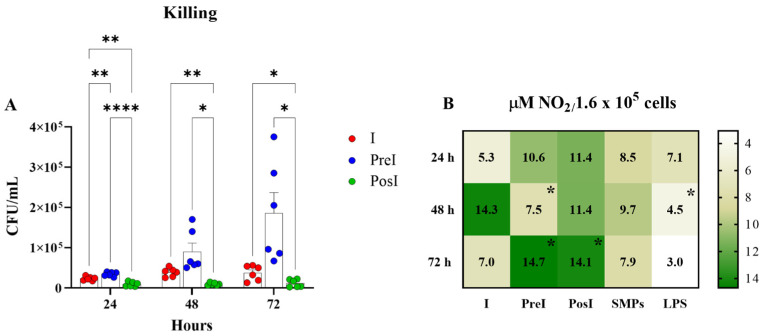
Mycobactericidal activity of MH-S macrophages under two different SMPs-addition strategies. (**A**). Killing of *M. tuberculosis*. Results are expressed as mean ± standard error of the mean (SEM) of three independent experiments. Significance was calculated with two-way ANOVA and Tukey’s multiple comparisons test. * *p* ≤ 0.5; ** *p* ≤ 0.1; **** *p* ≤ 0.001. (**B**). NO production. The heat map represents the mean ± SEM of three independent experiments. Significance was calculated with two-way ANOVA and Tukey’s multiple comparisons test. * *p* ≤ 0.5 regarding the I group at 48 h and regarding the LPS group at 72 h. I = Infected (MOI of 5); PreI = Pre-infection (SMPs added before infection); PosI = Post-infection (SMPs added after infection); LPS = *E. coli* lipopolysaccharide (1 μg/mL).

To determine whether the addition of SMPs affected the mycobactericidal performance of MH-S macrophages, CFU were calculated at 24, 48, and 72 h in all experimental groups. As shown in Figure 6A, the PosI group always had the lowest count of intracellular mycobacteria. On the contrary, at 48 and 72 h, the PreI group had the highest mycobacterial count, even more than the I group.

To correlate these data with NO production, we measured NO_2_ levels in supernatants from infected groups and compared them with those from groups stimulated with SMPs and LPS, the latter used as an inducer control. As shown in Figure 6B, the PosI group had the highest NO_2_ levels from the beginning (*p* ≤ 0.5 compared with the LPS group at 72 h), which agrees with the lowest CFU count in this group. Although no significant differences in NO_2_ production were observed between the PreI and PosI groups at 24 and 72 h, these levels were higher than those produced by the LPS-stimulated group. The I group showed the highest level of NO_2_ at 48 h (*p* ≤ 0.05) compared to the PreI and LPS groups; meanwhile, the group stimulated with SMPs showed a constant NO_2_ production that was not statistically different from the other groups but was higher than that of the LPS group. While there seems to be no correlation between NO_2_ levels and the elimination of mycobacteria, it is essential to maintain a consistent production rate to control their growth.

### 3.7. The SMPs-Addition Strategy Influenced the Metabolic Behavior of Infected Murine Macrophages

**Figure 7:**
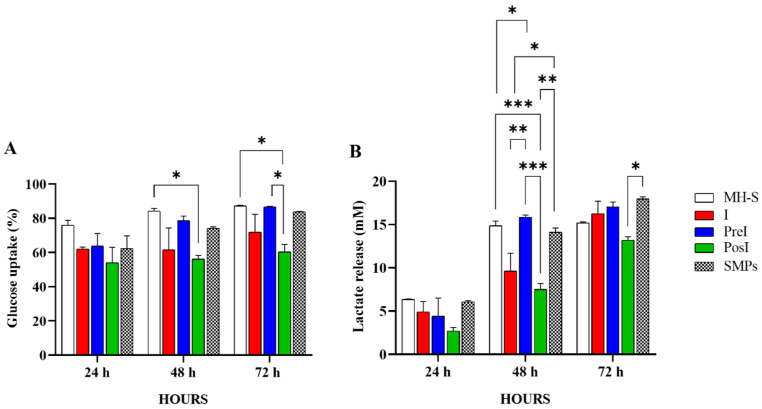
Metabolic behavior of MH-S murine alveolar macrophages infected with *M. tuberculosis* and treated with SMPs. (**A**)**.** Glucose uptake. (**B**)**.** Lactate release. Bars represent the mean ± standard error of the mean (SEM) of two independent experiments. Significance was calculated with two-way ANOVA and Tukey’s multiple comparisons test. * *p* ≤ 0.5; ** *p* ≤ 0.1; *** *p* ≤ 0.01. I = Infected (MOI of 5); PreI = Pre-infection (SMPs added before infection); PosI = Post-infection (SMPs added after infection).

Since glucose metabolism in macrophages plays a key role in mounting a robust immune response to mycobacterial infection, we compared the glucose uptake and lactate release in unstimulated, infected, and SMP-stimulated macrophages. As shown in Figure 7A, the PosI group, the one with the lowest bacillary count, had the lowest glucose uptake, with significant differences at 48 and 72 h compared with macrophages without stimulation and the PreI group (*p* ≤ 0.5). At all times, the PreI and SMPs groups had similar glucose uptake, which was not significantly different from that of macrophages without stimuli. As observed for glucose uptake, the lowest levels of lactate in the supernatant were detected in the PosI group (Figure 7B). At 48 h, the PosI group differed significantly from all other groups. Together, the results indicate that the addition of SMPs after infection did not pose a metabolic challenge to cells.

### 3.8. SMPs Modulated Cytokine/Chemokine Secretion, Without Causing an Exacerbated Proinflammatory Response in Infected Macrophages

**Figure 8:**
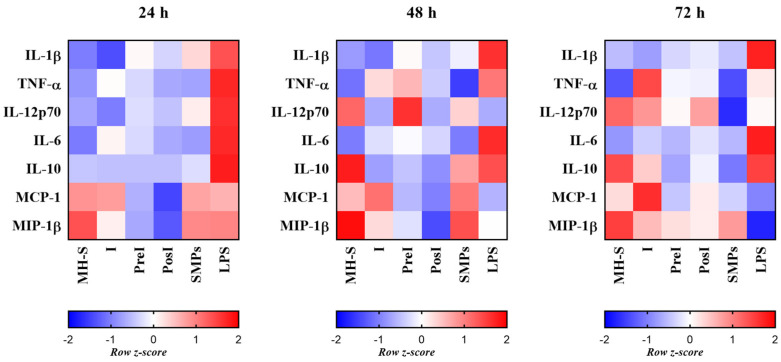
Cytokine profiles of MH-S macrophages infected and/or treated with SMPs. Cells without stimulation and stimulated with LPS are presented for comparison. Heatmap of cytokines normalized to row z-score. The white color corresponds to a z-score of 0, meaning the value equals the mean. Blue indicates negative z-scores (values below the mean), and red indicates positive z-scores (values above the mean). The saturation of the color indicates the magnitude of deviation from the mean—the farther from zero, the stronger the color.

We investigated whether the SMPs addition strategy affected cytokine production and whether these changes correlated with the mycobactericidal activity of MH-S macrophages. To this end, we quantified pro- and anti-inflammatory cytokines and the chemokines produced by all experimental groups. The production profile is presented as a heatmap, where lower (blue) and higher (red) values indicate concentrations relative to the group mean (Figure 8). Cytokine and chemokine concentrations (pg/mL) are presented in Figure S2.

Pro-inflammatory responses were predominantly induced by LPS stimulation. IL-1β increased significantly over time in that group, reaching its highest levels at 72 h (*p* ≤ 0.5). In contrast, the addition of SMPs alone did not induce production of this cytokine; it remained near the group mean and only marginally above it at 24 h. TNF-α production increased progressively in the I group, while higher levels were observed in the LPS group at 24 h and 48 h. In contrast, the PosI and SMPs groups exhibited the lowest TNF-α levels, suggesting that SMPs neither induce a TNF-α-mediated proinflammatory response on healthy macrophages nor potentiate infection-driven inflammation when added post-infection.

IL-12p70 showed only a slight increase at 24 and 48 h in macrophages stimulated with SMPs; however, the highest levels among infected groups were observed in the PreI group, which coincides with the loss of control of infection at this time point (Figure 6A). This suggests that adding particles before infection sensitizes macrophages, thereby enhancing IL-12p70 production after infection and compromising microbicidal activity.

IL-6 production was highest in LPS-stimulated control macrophages. In contrast, IL-6 remained consistently low over time in the group stimulated with SMPs (*p* ≤ 0.05). IL-10 was always elevated in the LPS group across all time points, consistent with previous reports in human alveolar macrophages, where LPS activates the phosphorylation of ERK, p38, and JNK MAP kinases, which are crucial for IL-10 expression in lung macrophages [37]. This cytokine also increased in the MH-S and SMPs groups at 48 h and reached higher levels in the I group at 72 h, possibly reflecting a compensatory macrophage response to regulate infection-induced inflammation.

Regarding chemokine production, MCP-1 increased over time in the I group and was moderately elevated in SMP-stimulated cells at 24 and 48 h. The PosI group, which displayed the lowest CFU count, showed a discrete increase in MCP-I over time. By contrast, MIP-1β levels were highest in the MH-S group, followed by the SMP-stimulated group, whereas the PreI and PosI groups exhibited only a slight increase at 72 h. Both chemokines declined over time in the LPS-stimulated group.

## 4. Discussion

In this work, we aimed to investigate whether exposure to SMPs could remodel or activate alveolar macrophages to enhance the innate immune response against *M. tuberculosis*. We based our inquiry on the possibility that SMPs, after intranasal administration, could reach the alveoli and impact both infected and surrounding macrophages, and also based on the consideration that it is possible to remodel alveolar macrophages to prepare them for response against infection, as occurs with vaccination [38].

We used the MH-S cell line, an immortalized murine alveolar macrophage line [39], which has been used as a model to study the interaction of *M. tuberculosis* with the host cell, and shows a comparable expression of surface markers and a similar capacity to interact with mycobacteria as primary alveolar macrophages [40,41].

Starch is a biocompatible polymer extensively used in the pharmaceutical and food industries. Accordingly, SMPs, which are not of microbial origin and do not contain extra antigenic molecules, are not expected to have intrinsic immunostimulant effects. However, innate immune responses in macrophages can be triggered simply by the process of phagocytosis, suggesting that free particles may condition and modulate immune responses [42,43].

In the context of infection with *M. tuberculosis*, several studies have documented that using particles to target infected macrophages is a good strategy to rescue them from the alternative activation state induced by mycobacteria to promote their survival, thereby switching towards a classical macrophage activation, favoring intracellular bacilli killing [3,4].

In this study, we first addressed concerns related to the safety of these particles. We explored the cytotoxic effects of high concentrations of particles (between 1 × 10^6^ and 10 × 10^6)^ on three macrophage cell lines and one epithelial cell line. We found a significant reduction in cell viability exclusively in the epithelial cell line (Figure 2). This difference is attributable to the cell types, since macrophages are professional APCs that routinely interact with microparticles and have developed mechanisms to phagocytose and eliminate them. This supports previous findings that nano- and microparticles can affect epithelial cell viability through oxidative stress and inflammatory responses [44,45].

Then, to assess the contribution of SMPs in known mechanisms that favor the elimination of *M. tuberculosis*, we evaluated their ability to induce ROS/RNS, apoptosis, and autophagy in non-infected macrophages [42,46,47]. The SMPs successfully induced ROS/RNS, while apoptosis was observed at minimal levels, and autophagy was not induced (Figure 3 and Figure 4).

Macrophage reactive oxygen species (ROS) play a critical role in phagocytosis, a fundamental process for eliminating pathogens and dead cells [48]. This is achieved through direct effects or indirect antimicrobial mechanisms, such as inflammasome activation and the release of cytokines and chemokines [49]. In Mtb-infected macrophages treated with SMPs, the production of these molecules may represent a contribution to microbicidal activity. However, it is also known that maintaining high levels of ROS may be detrimental to macrophages and lead to prolonged macrophage apoptosis [47,48]. In our study, this concern was dismissed because apoptosis in cells treated with SMPs was infrequent. This may be related to the lowest TNF-α levels in the SMP group, since the extrinsic apoptosis pathway can be activated when TNF-α interacts with the specific receptor TNF-R1 and adapter molecules, leading to caspase-8 activation [46]. Consequently, it can be suggested that SMPs-induced apoptosis may depend on the cytokine environment; further studies are therefore required to clarify this. Importantly, we did not observe necrotic cells, which is advantageous, as necrosis is a strategy that mycobacteria use to exit macrophages, evade host defenses, and spread.

Although some polymeric particles have been reported to effectively promote autophagy, enhancing the anti-mycobacterial activity in infected macrophages [4], we found no evidence of SMPs facilitating this mechanism. This observation, in turn, may help explain the limited production of IL-1β observed in the group stimulated only with SMPs. As documented, autophagy mediates the generation of non-signal peptide proinflammatory cytokines such as IL-1β by facilitating pro-IL-1β degradation [50].

Any direct antimycobacterial effect of SMPs was also ruled out. This was of particular interest because of reports describing the intrinsic antimycobacterial activity of some particles. Possible mechanisms involve particles directly interacting with the mycobacterial cell envelope, causing membrane damage and the subsequent extrusion of cytoplasmic material, ultimately leading to bacterial death [51]. However, using the REMA, we observed no mycobactericidal activity associated with the SMPs used in this study, indicating that the CFU reduction observed is a consequence of cellular activation, rather than a consequence of direct particle–bacterium interactions.

In the context of *M. tuberculosis* infection, regardless of the timing of SMPs addition (before or after infection), MH-S macrophages were capable of phagocytosing both particles and bacteria (Figure 5). Notably, membrane activation was observed in both phagocytosing and neighboring unstimulated macrophages, suggesting a role for paracrine signaling (Figure S1C).

Based on these observations, we investigated the microbicidal performance of MH-S macrophages under different particle-addition strategies and correlated these outcomes with additional indicators of cellular activation, including NO production, metabolic activity, and cytokine/chemokine production (Figure 6, Figure 7 and Figure 8).

The group that received the particles after infection (PosI) exhibited the lowest bacillary count, coinciding with the highest and sustained levels of NO, which plays a key role in mycobacterial killing during the first days of infection. Interestingly, there was no observable shift to aerobic glycolysis in MH-S macrophages in any treatment group (Figure 7).

This observation is important because macrophages typically switch to aerobic glycolysis in response to intracellular infections such as tuberculosis, leading to pro-inflammatory effector functions and increased production and secretion of lactate, which is accumulated in the lungs of infected animals or cells in culture, and is used by the mycobacteria as an additional carbon source to support their growth [52]. The group that received the particles after infection (PosI) exhibited the lowest bacillary count and the lowest glucose uptake and lactate release over time. This suggests that the addition of SMPs after infection may help control lactate production from the infection, thereby limiting the direct effects of lactate and restricting mycobacterial growth in this group.

As demonstrated by Maoldomhnaigh [53], the lactate released by infected macrophages induces metabolic changes in neighboring macrophages, characterized by reduced glycolysis, enhanced oxidative phosphorylation, and decreased production of proinflammatory cytokines such as IL-1β and TNF in infected macrophages, which affects the subsequent responses to *M. tuberculosis* [54]. This, however, needs to be carefully interpreted and thoroughly investigated, as numerous factors involved in the metabolic response of macrophages to mycobacterial infection are not accounted for in vitro assays.

Finally, we aimed to correlate the previous data with the production of certain cytokines involved in the response to mycobacterial infections. It is well attested that the cytokine profile of alveolar macrophages infected with *M. tuberculosis* exhibits a complex response that can be both pro-inflammatory and anti-inflammatory. Several factors, including prior pathogen exposure and the local microenvironment, influence this balance [38]. Additionally, alveolar macrophages play a crucial role in maintaining lung homeostasis and tolerance to inhaled antigens. Therefore, their immune response is directed to control inflammation, which is critical for the infection resolution while preventing tissue damage [38].

In this study, stimulation with SMPs had a limited effect on the cytokine profile of alveolar macrophages. As observed in Figure 8, exposure to SMPs resulted in reduced levels of IL-1β and IL-12p70 compared to other groups, indicating that, in the absence of infection and signals from other immune or epithelial cells, these particles have a limited proinflammatory effect. Previous in vitro studies using starch-based polymers to stimulate mononuclear cells have reported diverse outcomes, including high levels of IL-6 and TNF-α, and very low or undetectable levels of IL-1β [25]. In our case, the SMPs did not induce IL-1β, IL-6, or TNF-α, which suggests that SMPs have a lower proinflammatory effect than other starch-based polymers and composites.

Consistent with the best mycobactericidal performance observed in infected macrophages treated with the particles (PosI group), with significant reductions in chemokines like MCP-1 and MIP-1β, which are important for immune cell recruitment and granuloma formation. This is noteworthy, as MCP-1 is associated with tuberculosis pathogenesis and severity, particularly with hypervirulent *M. tuberculosis* strains [54,55,56]. Consequently, modulating chemokine production in infected macrophages through particle-based interventions may be a promising strategy to overcome immunosuppressive signals generated by the pathogen, thus preventing the deactivation of macrophages.

On the contrary, in the group with the highest CFU count, the PreI group, we observed higher levels of TNF-α and IL-12p70 at 48 h, which indicates that the addition of SMPs before infection may amplify the proinflammatory response induced by infection itself. While these findings may guide a rational use of SMPs in the context of *M. tuberculosis* infection, they only provide a partial view of the immune landscape. Therefore, a more detailed scrutiny of the cytokine profile produced by these particles is required, since it is known that some types of particles, such as poly (lactic-co-glycolic acid) (PLGA) particles, have been demonstrated to reduce bacillary viability in infected macrophages without major alterations in cytokine secretion profiles [4].

## 5. Conclusions

Searching and identifying new molecules that can reprogram macrophage capacities for killing *M. tuberculosis* is considered a promising strategy to counteract the limitations of current TB treatment, in terms of safety, efficacy, and side effects, which are also linked to the emergence of MDR mycobacteria. Ideally, the new treatment alternatives must be for intranasal or pulmonary delivery. However, important challenges exist in developing these systems because they must cross the nasal mucosa, contend with mucociliary elimination, and reach the alveoli in the lungs to ultimately contribute to innate immune responses, primarily mediated by alveolar macrophages. Therefore, nano- and microparticles are considered a good alternative due to their advantages in contending with these challenges and their potential to induce and modulate the immune response.

In this study, we demonstrated that starch microparticles (SMPs) can modulate the microbicidal capacity of murine alveolar macrophages during *M. tuberculosis* infection, depending on the particle-addition strategy, without acting as direct antimycobacterial agents. SMPs were confirmed to be biocompatible with macrophages, inducing no evident cytotoxicity, necrosis, or excessive apoptosis, while producing controlled ROS/RNS upon phagocytosis. These SMPs, without a drug or antigen payload, showed efficacy in limiting intracellular mycobacteria replication when added as therapy to previously infected macrophages (PosI group). They induced autophagy-independent killing of bacilli and modulated macrophage cytokine/chemokine secretion without exacerbating the proinflammatory response. We associated mycobacterial killing by infected macrophages with membrane activation and paracrine signaling effects extending to neighboring cells.

The findings demonstrate that SMPs effectively fine-tune macrophage responses to enhance intracellular killing while preventing excessive inflammation. This research underscores the promising potential of biocompatible particle-based strategies as adjunct approaches to enhance host control of *M. tuberculosis*, thereby expanding the therapeutic spectrum of these particles after intranasal or pulmonary administration.

## Figures and Tables

**Table 1:** Anti-*M. tuberculosis* effects and cytotoxicity levels of SMPs.

Compound	MIC_100_ (μg/mL)	IC_50_ in MH-S Cells (μg/mL)	IC_50_ in RAW264.7 Cells (μg/mL)	IC_50_ in THP-1 Cells (μg/mL)	IC_50_ in Vero Cells (μg/mL)	Selectivity Index (SI)
SMPs	>500	>1000	>1000	>1000	>1000	>2
RIFAMPICINE	0.06	>1000	ND	ND	ND	>16,666

MIC = minimal inhibitory concentration determined by using REMA; IC_50 =_ inhibitory concentration 50; SI = Selectivity index = IC_50_/MIC_100_. SMPs = starch microparticles; ND = not determined.

## Data Availability

The original contributions presented in this study are included in the article/Supplementary Materials. Further inquiries can be directed to the corresponding authors.

## Supplementary Materials

The following supporting information can be downloaded at: https://www.mdpi.com/article/10.3390/microorganisms14040800/s1, Figure S1: Representative transmission electron micrographs showing MH-S murine alveolar macrophages infected with M. tuberculosis or treated with SMPs; Figure S2: Cytokine and chemokine production of MH-S murine alveolar macrophages infected with Mtb or treated with SMPs.

## Abbreviations

The following abbreviations are used in this manuscript:

TB	tuberculosis
BCG	bacillus Calmette–Guérin
SMPs	starch microparticles
PLA	poly (L-lactic acid)
PLGA	poly (Lactic-co-glycolic-acid)
ROS/RNS	oxygen and/or nitrogen reactive species
CFUs	colony-forming units
RPMI	Roswell Park Memorial Institute
FBS	fetal bovine serum
DMSO	dimethyl sulfoxide
MTT	(3-(4,5-dimethylthiazol-2-yl)-2,5-diphenyltetrazolium bromide)
PMA	phorbol 12-myristate-13-acetate
NO	nitric oxide
IC_50_	inhibitory concentration 50
TEM	transmission electron microscopy
I	infected
PreI	pre-infection
PosI	post-infection
LPS	lipopolysaccharide
