# Supplementary material for: Effect of Starch Microparticles on the Activation and Bactericidal Activity of Murine Alveolar Macrophages Infected with Mycobacterium tuberculosis
Source: Microorganisms. 2026 Apr 1;14(4):800. doi: 10.3390/microorganisms14040800 (PMC13118927; doi:10.3390/microorganisms14040800)
Supplement: Supplementary file 1 [file microorganisms-14-00800-s001.zip › microorganisms-4189682-supplementary.pdf]

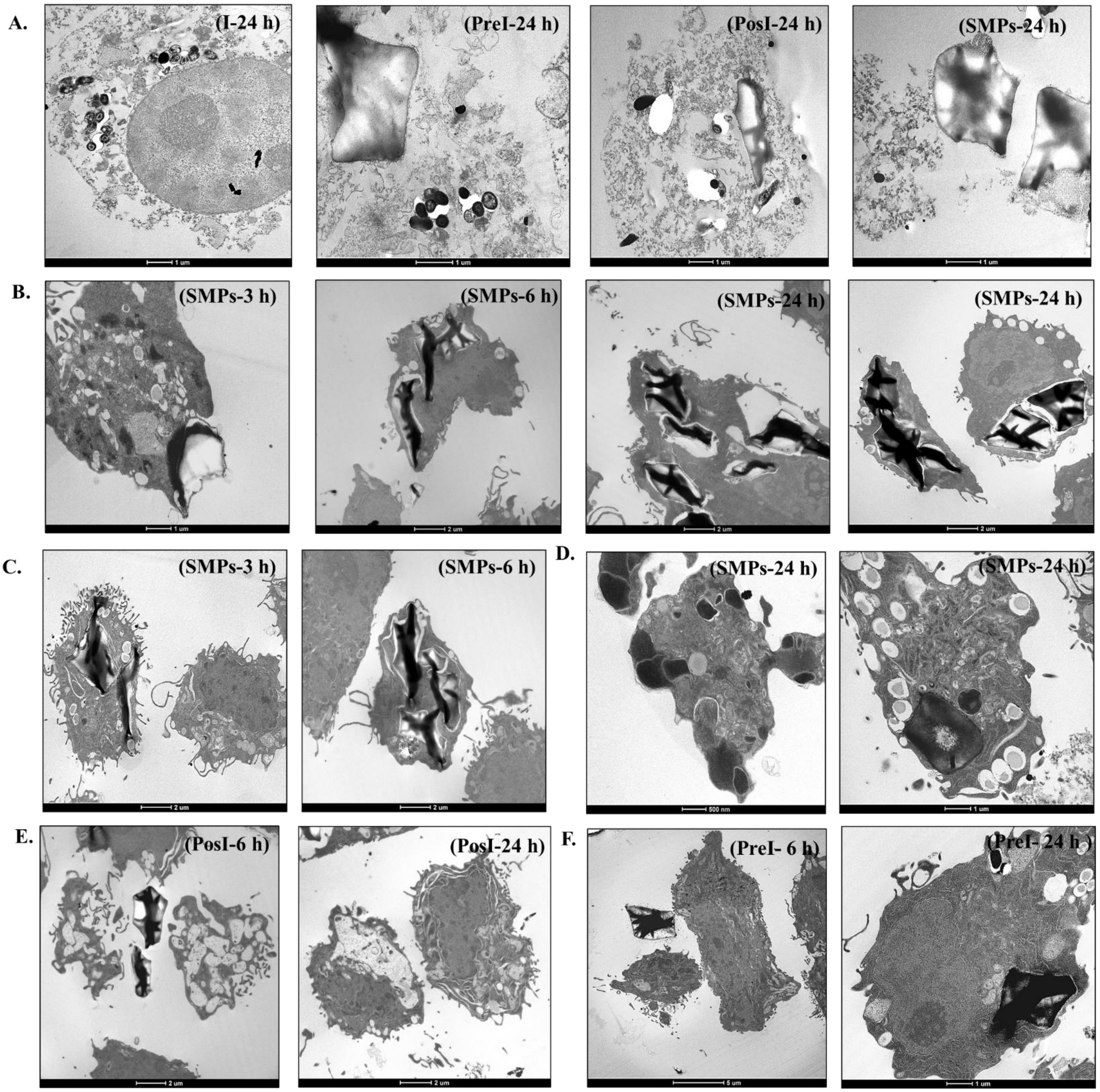

Figure S1. Representative transmission electron micrographs showing MH-S murine alveolar macrophages infected with *M. tuberculosis* or treated with SMPs. **A**. Dying cells and digested bacilli and particles at 24 hours of incubation in the (I), (PreI), (PosI), and (SMPs) groups. **B.** Macrophages of the (SMPs) group, phagocytosing and several particles at 6 and 24 hours of incubation. **C.** Macrophages of the (SMPs) group showing empty activated macrophages in proximity to phagocytosing cells at 3 and 6 hours of incubation. **D.** Macrophages of the (SMPs) undergoing apoptosis at 24 hours of incubation. **E.** Macrophages of the (PosI) group, showing empty activated macrophages in proximity to phagocytosing cells at 6 and 24 hours of incubation. **F.** Macrophages of the (PreI) group, showing no dramatic activation patterns, nor in proximity with particles nor phagocytosing, at 6 and 24 hours of incubation.

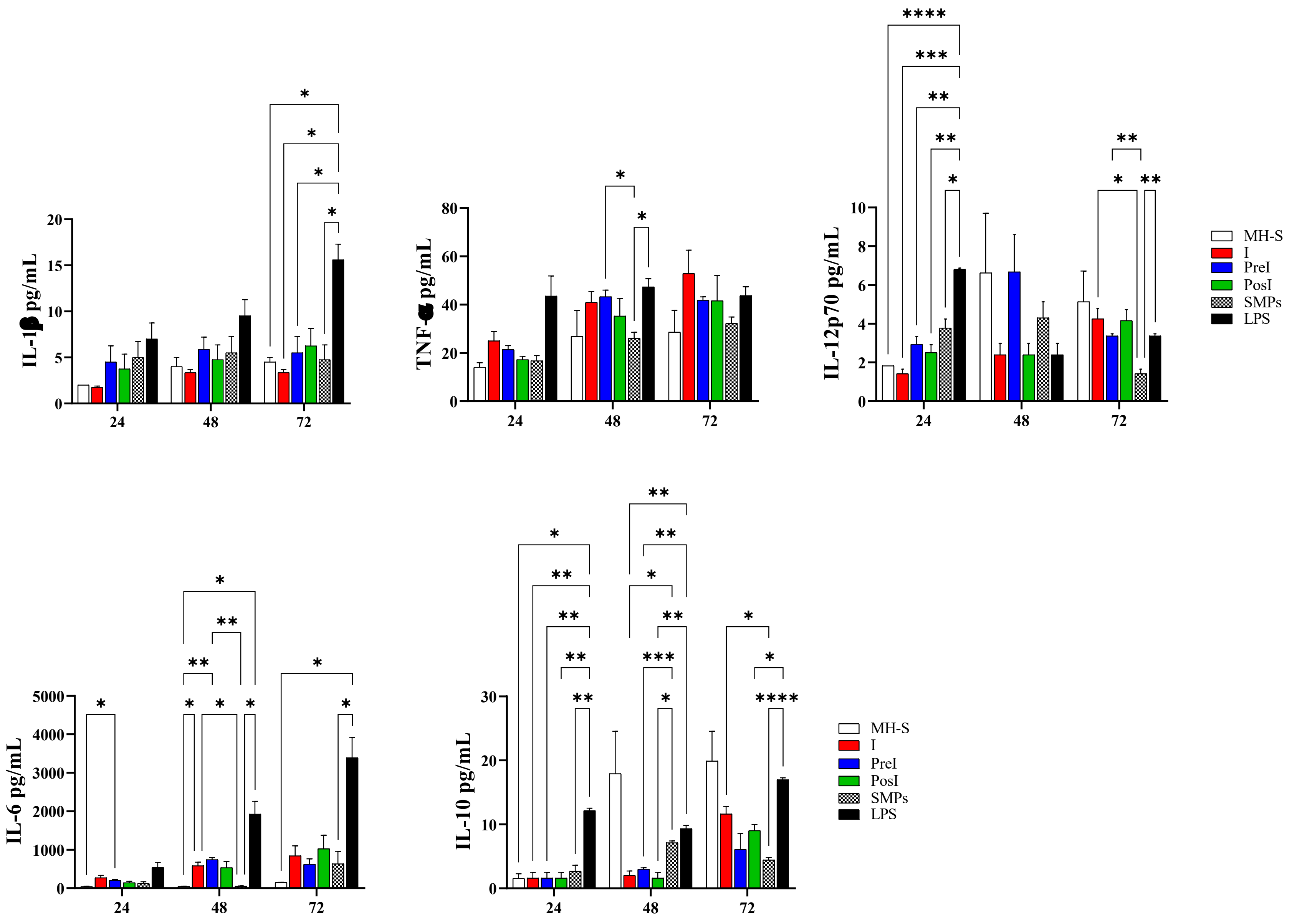
IL-1β pg/mL
0
5
10
15
20
24
48
72
*
*
*
*
TNF-α pg/mL
0
20
40
60
80
24
48
72
*
*
IL-12p70 pg/mL
0
2
4
6
8
10
24
48
72
****
***
**
**
*
**
*
**
MH-S
I
PreI
PosI
SMPs
LPS
IL-6 pg/mL
0
1000
2000
3000
4000
5000
24
48
72
*
*
**
**
*
*
*
*
*
*
IL-10 pg/mL
0
10
20
30
24
48
72
*
**
**
**
**
**
**
*
**
***
*
*
*
****
MH-S
I
PreI
PosI
SMPs
LPS

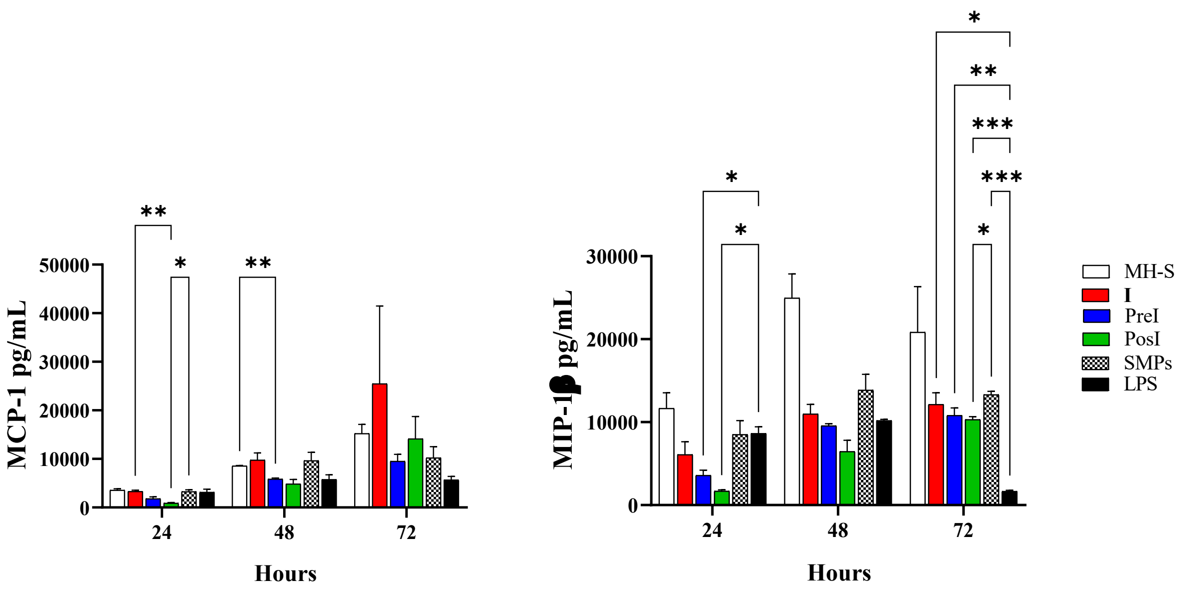

Figure S2. Cytokine and chemokine production of MH-S murine alveolar macrophages infected with Mtb or treated with SMPs. Bars represent the mean ± standard error of the mean (SEM) of cytokines or chemokines (pg/ml) detected in supernatants of three independent experiments. Significance was calculated with two-way ANOVA and Tukey's multiple comparisons test. *$p \leq 0.05$; **$p \leq 0.01$; ***$p \leq 0.001$; ****$p \leq 0.0001$. **I** = Infected with Mtb (MOI of 5); **PreI** = Pre-infection, SMPs added before infection; **PosI** = Post-infection, SMPs added after infection.
